# Efficacy of MI Paste® on Bleaching-Related Sensitivity: Randomized Clinical Trial

**DOI:** 10.1155/2021/9963823

**Published:** 2021-06-14

**Authors:** Shaista Rashid, Mohamed ElSalhy

**Affiliations:** ^1^A. T. Still University-Missouri School of Dentistry & Oral Health, St. Louis, Missouri, USA; ^2^College of Dental Medicine, University of New England, Portland, Maine, USA

## Abstract

**Background:**

To evaluate the effectiveness of MI Paste® in reducing sensitivity associated with vital tooth bleaching.

**Methods:**

This randomized controlled split-mouth clinical trial included 45 subjects that were randomly divided into two groups. In Group 1, the maxillary arch was the control arch (only bleaching), while the mandibular arch was the intervention arch (bleaching and MI Paste®). In Group 2, the mandibular arch was the control arch (only bleaching), while the maxillary arch was the intervention arch (bleaching and MI Paste®). Subjects started with the control arch and then switched to the intervention arch after two weeks. Subjects were instructed to use MI Paste® in a custom tray for 5 minutes, wait for 1 hour, and then bleach overnight using a different tray. Sensitivity was measured using both a thermal sensitivity test and a daily log of sensitivity for 14 days. Shade was evaluated using a colorimeter and a shade guide.

**Results:**

Immediately after treatment, the thermal test sensitivity scores for the arches bleached without MI Paste® were greater than those with MI Paste® (*p*=0.011). Arches not receiving the MI Paste® treatment showed significantly higher VAS sensitivity scores during the 14-day period of bleaching (*p*=0.002). The mean score for the 14-day period was 37.9 for the arches not treated with MI Paste® versus 27.5 for the treated arches. Both the intervention group and the control group showed significantly lighter shade relative to baseline (*p* < 0.001) with no significant difference between them (*p*=0.42).

**Conclusion:**

MI Paste® significantly reduced the sensitivity associated with bleaching and did not interfere with shade change.

## 1. Introduction

Tooth whitening is currently one of the most conservative, noninvasive methods of altering the shade of vital teeth. Vital bleaching can be dentist-supervised in office or at home and/or self-administered over the counter [1]. Side effects associated with vital bleaching include mucosal irritation and thermal sensitivity [2–4]. A systematic review of bleaching-induced sensitivity reported significant variation in duration and severity of sensitivity [5]. Studies have reported the prevalence of thermal sensitivity associated with vital bleaching varies from 0% to 100% [6–11].

The primary cause of thermal sensitivity after bleaching is believed to be the exposure of dentinal tubules [7, 8, 12, 13]. Several products have been marketed in an attempt to reduce sensitivity associated with bleaching. The main ingredients of these products include potassium nitrate and/or fluoride [12–15]. Fluoride is believed to decrease sensitivity by blocking the dentinal tubules and reducing the fluid flow to the pulp, while potassium nitrate is believed to reduce sensitivity by reducing the ability of the nerve to repolarize after initial depolarization caused by pain sensation [14, 16]. Other new formulas containing milk-derived proteins reduce sensitivity by occluding the dentinal tubules [17].

MI Paste® is a desensitizing agent approved by the FDA for treating tooth hypersensitivity. The active ingredient in MI Paste® is the milk-derived casein phosphopeptide-amorphous calcium phosphate (CPP-ACP). Calcium phosphate is insoluble at neutral pH; however, CPP maintains the calcium and phosphate in an amorphous crystalline state. In the oral cavity, CPP parts attach to the biofilm and release calcium, phosphate, and hydroxide ions at the tooth surface, leading to remineralization [17]. CPP-ACP also causes rapid desensitizing effect by immediate protein binding followed by precipitation and deposition of calcium phosphate compound onto the dentin surface, leading to occlusion of dentin tubules [18]. Few clinical trials showed CPP-ACP to be effective in reducing sensitivity related to in-office bleaching when applied topically either by dental professionals or by patients. A randomized clinical trial reported a single 10-minute professional application of CPP-ACP leads to a significant reduction in postoperative sensitivity following the in-office bleaching [19]. Another study reported a reduction in intensity and duration of sensitivity with chewing gum containing CPP-ACP following in-office tooth whitening [20].

In contrast to the available literature on in-office bleaching-related sensitivity, only one clinical trial has reported CPP-ACP (MI Paste®) effectiveness in reducing at-home bleaching-related sensitivity [21]. The trial reported only initial sensitivity relief with MI Paste® at Day 3 of the 14-day at-home bleaching cycle. Possible cross-contamination between the bleach and MI Paste® in the trial can be a potential reason for the lack of MI Paste® effectiveness. Therefore, this study aimed to evaluate the efficacy of MI Paste® in reducing bleaching-related sensitivity among patients undergoing at-home bleaching and its effect on color change.

## 2. Material and Methods

### 2.1. Study Design

This was a randomized, controlled, split-mouth crossover design clinical trial. Subjects were randomized into two groups depending on the control and intervention arches by the principal investigator using simple random allocation. The control arch received only bleaching, while the intervention arch received bleaching and PROSPEC MI Paste® (GC American Inc, Alsip, IL USA).

- Group 1: the maxillary arch was the control arch (only bleaching), while the mandibular arch (MN) was an intervention arch (bleaching and MI Paste®)
- Group 2: the mandibular arch was the control arch (only bleaching), while the maxillary arch (MX) was the intervention arch (bleaching and MI Paste®)

Ethical permission for the study was obtained from the University Intuitional Review Board (No. 200604706). The study was conducted in full accordance with the Helsinki Declaration and reported using CONSORT guidelines. The trial was registered with clinicalTrials.gov Trial Registration Number: NCT04112706 (https://clinicaltrials.gov/ct2/show/NCT04112706). Informed consent was obtained from subjects prior to their enrollment in the study.

### 2.2. Sample Size Calculation

The sample size was calculated to be 32 individuals (16 individuals per group). The calculation was based on an alpha of 0.05, power of 80%, the expected difference in means of 10%, and a standard deviation of 10%. As dropout and noncompliance with the protocol of 40% was expected, a total of 46 individuals were targeted.

### 2.3. Participants' Inclusion and Exclusion Criteria

Forty-six participants were recruited at the Oral Health Center. Participants were included in the study if they were between 18 and 55 years old, had no prior history of bleaching, were not using any desensitizing agents, and had anterior teeth discoloration of shade A2 or darker Vita Classical™ shade guide (Vita North America, Yorba Linda, CA, USA). Participants were excluded from the study if they were allergic to milk protein, pregnant, or taking NSAIDs; had crown or restorations on an anterior tooth; and had a gingival recession, periodontal disease, and scaling or periodontal surgery performed in the past six months.

### 2.4. Intervention

The GC TiON™ Tooth Whitening Take Home kit (GC American Inc, Alsip, IL, USA) was used in the study. The TiON™ system included 15% carbamide peroxide gel and PROSPEC MI Paste®. Custom trays with reservoirs were made for both bleaching gel and MI Paste® delivery. To avoid contamination, the control arch was bleached first. Both groups were instructed to bleach the control arch first using 15% carbamide peroxide in custom scalloped trays. Group 1 subjects were instructed to bleach the maxillary arch, while Group 2 subjects were instructed to bleach the mandibular arch. Participants were instructed to floss and brush their teeth and then wear the trays with bleaching gel at night for 6–8 hours for two weeks. Subjects were given a Visual Analogue Scale (VAS) daily log to document sensitivity for each day during the bleaching period of two weeks.

**Figure 1:**
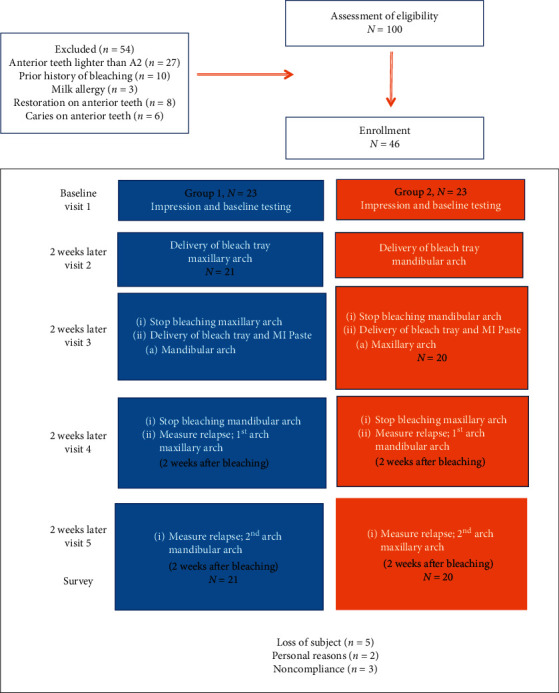
Description of intervention.

After two weeks, subjects stopped bleaching on their respective arches and were given bleach and MI Paste® for the opposing arch. Participants were instructed not to bleach the first arch anymore and were given two trays for the second arch (intervention arch). Participants were instructed to floss and brush first and then load the nonscalloped tray with MI Paste®, wear it for 5 minutes, remove the tray, spit out the excess, and instructed not to eat or drink for one hour. Participants were instructed not to rinse their mouth or brush their teeth again after the wait time of one hour. They were instructed to bleach the arch with the second scalloped tray for 6–8 hours at night for the next two weeks (Group 1 bleach/MI Paste® on the mandibular arch, and Group 2 bleach/MI Paste® on the maxillary arch). Participants were given another Visual Analogue Scale (VAS) daily log to document the sensitivity for each day during the second bleaching period. A flowchart with a detailed description of the intervention is shown in Figure 1.

### 2.5. Measurements

#### 2.5.1. Sensitivity

Sensitivity was measured using both thermal sensitivity test and longitudinal Visual Analogue Scale (VAS) daily log. The thermal sensitivity test was measured by using a 1-second air blast at 70°F from the dental unit air syringe as per the American Dental Association (ADA) guidelines. Both the maxillary and mandibular anterior teeth (#6–11 and #22–27) were evaluated for sensitivity. A scale of 0–3 was used to measure the pain response, with 0 indicating “no pain” and 3 indicating the “severe pain” that lasted for more than ten seconds [18]. Thermal sensitivity measurements were made at baseline (Time 0), two weeks after initiation of bleaching (Time 1), and two weeks after the end of treatment (i.e., four weeks after initiation of bleaching; Time 2) for each arch.

Sensitivity was also measured using the longitudinal Visual Analogue Scale for the sensitivity of 0–10, with 0 being “no pain” and 10 being “severe pain.” Subjects were asked to pick a number. The VAS for sensitivity was assessed at baseline (Day 0) and daily during the two weeks of bleaching (Days 1 through 14). Subjects were instructed not to take analgesic during the course of study.

#### 2.5.2. Shade

The Vita Classical™ shade guide (Vita North America, Yorba Linda, CA, USA) was used to determine the shade of the teeth under standardized conditions for color corrected light. Vita Classical™ shade scores were ordered from 1 to 16 according to the lightness grouping recommended by the manufactures.

In addition, the shade was evaluated digitally with a handheld colorimeter “Shade Vision” (X-rite Inc, Grand Rapids, MI, USA). Shade Vision identifies color differences using the three-dimensional CIE *L*^*∗*^*a*^*∗*^*b*^*∗*^ values system. The *L*^*∗*^ values represented color gradients from white to black and were used in this study. For the *L*^*∗*^ measurement in the study, the whiter color had higher reading, while the darker color had a lower reading.

Shade measurements were made at baseline (Time 0), two weeks after initiation of bleaching (Time 1), and two weeks after the end of treatment (i.e., four weeks after initiation of bleaching; Time 2) for each arch. Shade was taken on maxillary and mandibular anterior teeth (#6–11 and #22–27). The operator was not blinded to the treatment group.

#### 2.5.3. Participants' Survey

Participants were given a survey at the end of the study, examining their perception of the ease of application of MI Paste® as well as the impact of MI Paste® on sensitivity and gingival inflammation.

### 2.6. Data Analysis

Data were managed and analyzed using SPSS 22.0 software (IBM Corp., Armonk, NY, USA). Frequencies and percentages were used to describe categorical variables. Mean and standard deviation were used to describe continuous variables. The difference in mean scores with groups and between groups was evaluated using the *t*-test. Outcomes used to compare intervention and control groups were the overall (arch-specific) VAS sensitivity scores, daily longitudinal VAS scores, the Vita shade match, and the CIELAB *L*^*∗*^*a*^*∗*^*b*^*∗*^ assessments (lightness).

## 3. Results

Forty-one out of 46 participants completed the study. Five subjects dropped out during the study; two for personal reasons and three were noncompliant with the protocol. The study included 23 females and 18 males. Age ranged from 21 to 52 years, with an average age of 36.7 years. All the 41 participants attended the recall visits and completed the daily log as well as the poststudy survey. No participants reported any harm.

### 3.1. Sensitivity

#### 3.1.1. Thermal Sensitivity Scores

There was no significant difference between treated or untreated arches in the thermal sensitivity scores at baseline (*p*=0.063). Immediately after treatment (Time 1), the thermal sensitivity scores for the arches bleached without MI Paste® were greater than those with MI Paste® (*p*=0.011). There was no significant difference in the thermal sensitivity scores at the two weeks' visit (*p*=0.214) (Table 1).

#### 3.1.2. Longitudinal Visual Analogue Scale Sensitivity Scores

**Figure 2:**
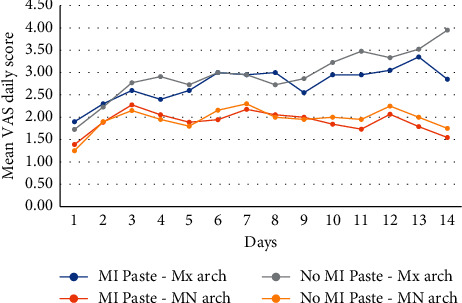
Daily changes in VAS scores.

During the 14-day period of bleaching, arches bleached MI Paste® showed significantly lower mean total VAS sensitivity scores than the arches bleached without MI Paste® (*p*=0.002). The mean score for the 14-day period was 37.9 for the control versus 27.5 for the MI Paste® arches (Table 2). When comparing daily scores, MI Paste®-treated maxillary arches showed significantly lower sensitivity scores starting at Day 9 than maxillary arches bleached without MI Paste®, while mandibular arches showed significant difference starting at Day 10 (*p* < 0.05). Daily changes in the VAS scores are shown in Figure 2.

### 3.2. Shade Analysis

#### 3.2.1. CIELAB *L*^∗^*a*^∗^*b*^∗^ Color Scale: Lightness Dimension

**Figure 3:**
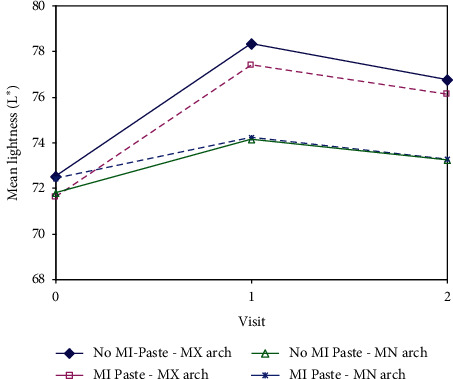
Average lightness value over time.

There was no significant difference in the lightness scores at baseline between the arches that were to receive MI Paste vs. the control arches (mean = 72.1 vs. 72.1; *p*=0.69), as well as no significant difference between maxillary and mandibular arches (*p*=0.81). Both arches treated with MI Paste® and those bleached without MI Paste® showed significant shade change relative to baseline regardless of the arch allocation group (all *p* values were <0.01). The shade change was significantly higher in the maxillary arch than the mandibular arch in both groups (*p*=0.010). Lightness scores at baseline, Time 1, and Time 2 for treatment and control arches are summarized in Table 3. Average lightness values in different arches are shown in Figure 3.

#### 3.2.2. Vita Shade Match

Arches treated with MI Paste®, as well as those bleached without MI Paste®, showed significant net shade change (*p* < 0.0001) compared with baseline with no significant difference between the treatment and the control group (*p*=0.42).

### 3.3. Participant Experience Survey

**Figure 4:**
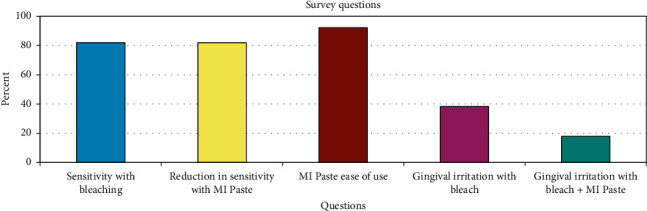
Survey questions.

About 80% of participants experienced bleaching-related sensitivity, and 38% experienced gingival irritation. Ninety-two percent of the subjects felt that MI Paste® was easy to use. About 80% of participants reported a reduction in sensitivity, and 18% reported a reduction in gingival irritation with MI Paste® use (Figure 4).

## 4. Discussion

This study aimed to evaluate the effect of MI Paste® in reducing bleaching-related sensitivity and its impact on color change. MI Paste® used during bleaching was associated with significantly lower sensitivity. The addition of MI Paste® into the treatment regime did not affect the whitening efficacy.

Several products have been marketed in an attempt to reduce sensitivity associated with bleaching. These products include therapies applied at the dental office and those applied at home by patients. Two treatment approaches used for treating the dentinal hypersensitivity are tubular occlusion and nerve depolarizing [20, 22–25]. Tubular occluding agents like stannous fluoride (SnF_2_), arginine, and strontium salts block exposed dentinal tubules, while nerve depolarizing agents like potassium ions interfere with neural transduction of pain stimuli [26–28]. Adding these agents to dentifrices showed effectiveness against sensitivity [25–27]. CPP-ACP is the main active ingredient in MI Paste® [17]. CPP-ACP crystals of MI Paste® fill in the microscopic enamel surface defects and make teeth smoother, stronger, and less sensitive [29–31]. In addition, MI Paste® was proposed to promote remineralization of enamel, which can help in reducing sensitivity. Microscopic analysis of samples bleached with 40% hydrogen peroxide and treated with CPP-ACP showed amorphous crystal deposition on the surface, implying remineralization [3]. MI Paste® also contains glycerol, which is added to increase the paste smoothness and viscosity. Glycerol is a humectant (i.e., it absorbs water and keeps the teeth moist by sticking to the enamel surface), which may prevent the initial dehydration caused by bleaching. It was proposed that glycerol plug is the initial mechanism preventing tooth sensitivity followed by the calcium and phosphate ion precipitation leading to tubular occlusion, thus sustaining the benefit of MI Paste® in reducing sensitivity [17].

The impact of CPP-ACP on postbleaching sensitivity has been documented after in-office bleaching. CPP-ACP showed a significant reduction in sensitivity compared with sodium fluoride after in-office bleaching with 40% carbamide peroxide [32]. However, CPP-ACP had some impact on shade stability [32].On the other hand, Alexandrino et al. reported a significant reduction in sensitivity with the combination of CPP-ACPF with 35% hydrogen peroxide with no effect on color change [33]. In vivo studies using the CPP-ACP along with bleaching have shown no interference with color change and improved the enamel irregularities and porosities, which can explain the reduction in sensitivity [34–36]. This study supports previous reports that CPP-ACP is effective in reducing sensitivity with no impact on color change.

At-home bleaching used in combination with MI Paste® was only evaluated in one previous study. The study assessed the impact of MI Paste® on tooth sensitivity and color change when combined with at-home bleaching [21]. In that study, patients were instructed to use bleaching gel in custom trays for four hours and then use MI Paste® in the same tray for 30 minutes. The results of the study showed a reduction in sensitivity only on Day 3 of the bleaching when compared with placebo with no difference on Day 7 and Day 14 [21]. In our study, reduction in sensitivity with the use of MI Paste® over the bleaching period of two weeks was seen, even though bleaching was used for an extended period of time (6–8 hr) and MI Paste® was applied for a shorter duration (5 minutes). One possible explanation could be that different trays for bleach and MI Paste® were used in this study, which decreases the risk of cross-contamination and may yield better results. In addition, sensitivity was only reported by a Visual Analogue Scale log in the previous study, while this study used multiple ways for evaluating sensitivity with similar results. More studies are needed to confirm the impact of MI Paste® on reducing at-home bleaching-related sensitivity.

Since there were no clear recommendations about the application of MI Paste® available at the time of the study, only one application of MI Paste® was used before bleaching treatment in our study. The manufacturer's instructions were to apply MI Paste® with a finger or in a tray for 5 minutes before and after bleaching. In this in vivo study, MI Paste® was applied in the custom tray for 5 minutes only prior to bleaching to increase subject compliance. This is one of the limitations of the present study. If the manufacturer's recommendations were followed and the second application of MI Paste® after bleaching was applied, better results may have been reported. In addition, due to a lack of a clinical protocol on how much time gap between the MI Paste® application and the bleaching treatment, one-hour wait time was used in the study to control any possible interaction between MI Paste® and bleaching gel. This wait time was described by the patient as a major inconvenience. As no interaction of the MI Paste® with the bleaching gel was found in the present study, future studies may try a shorter waiting period.

A limitation of the study is the lack of placebo control. Placebo was not used due to the inability to manufacture an inactive formulation and limited resources. Studies have shown a 20%–30% reduction in sensitivity can be attributed to the placebo effect [35]. The presence of the placebo group may have increased the reliability of this study. In addition, only the thermal sensitivity test and daily VAS were used to evaluate sensitivity. Using other methods like tactile or chemical testing may give different results. However, most sensitivity-related clinical studies have used the VAS and thermal scales similar to this study. In addition, the efficacy of color change was measured by using only the *L*^∗^ values of CIE *L*^∗^*a*^∗^*b*^∗^ as the goal was to evaluate the change in lightness. Evaluating the total color difference (∆*E*) may help understand the shade stability more. Another limitation of the study is that gingival inflammation was self-reported rather than a standardized objective measure of gingival irritation.

## 5. Conclusion

In conclusion, the use of MI Paste® together with bleaching significantly reduced the bleaching-associated sensitivity and gingival inflammation with no impact on shade change. MI Paste® can be a simple and cost-effective treatment modality for at-home bleaching-related sensitivity.

## Figures and Tables

**Table 1:** Mean (SD) thermal sensitivity scores at each time point for treatment and control arches.

VAS scores	Time of measurement					
	Baseline		Immediately after treatment		2 weeks after end of treatment	
	Mean	SD	Mean	SD	Mean	SD
With MI Paste (treatment)	0.675	0.971	2.050	1.782	0.600	0.928
Without MI Paste (control)	0.975	1.230	2.900	1.411	0.825	1.010
*p* value	0.063		0.011		0.214	

**Table 2:** Mean (SD) visual analogue scale sensitivity scores of 14 days for treatment and control arches.

Variable	Mean	Std. dev	*p* value
With MI Paste (treatment)	27.52	18.61	0.002
Without MI Paste (control)	37.95	16.16	

**Table 3:** Lightness scores at baseline, Time 1, and Time 2 for treatment and control arches.

Time	Baseline		Time 1		Time 2	
Measures	Treatment	Control	Treatment	Control	Treatment	Control
Mean	71.99	72.13	75.89	76.13	74.78	74.91
Std. dev	2.67	2.60	3.42	3.35	3.11	3.13
*p* value	0.690		0.752		0.853	

## Data Availability

The data used to support the findings of this study are available from the corresponding author upon request.
